# Accuracy of Quantifying Hypotension During Surgery Using Physiological Sensor Data

**DOI:** 10.1109/JTEHM.2026.3698197

**Published:** 2026-05-29

**Authors:** Martin Jacobsson, Arman Valadkhani, Greg Winski, Max Bell

**Affiliations:** Department of Biomedical Engineering and Health SystemsKTH Royal Institute of Technology 100 44 Stockholm Sweden; Perioperative Medicine and Intensive Care (PMI)Karolinska University Hospital 171 76 Stockholm Sweden; Department of Pharmacology and PhysiologyKarolinska Institutet 27106 171 77 Stockholm Sweden; CLINTECKarolinska Institutet 27106 171 77 Stockholm Sweden

**Keywords:** Arterial blood pressure, downsampling, hypotension, mean arterial blood pressure

## Abstract

Objective: During surgery it is common to measure the arterial blood pressure. One important reason is to monitor for hypotension, a too low blood pressure, which is known to be harmful. Since post-surgical complications correlate with hypotension, it is common to calculate the number of hypotensive events, their cumulative length, or their cumulative area under threshold. To make the data collection process manageable, parameter data is often stored only once every 15 seconds and then used to derive the amount of hypotension.Methods and procedures: In this study, we calculate the errors introduced in quantizing hypotension based on different amount of downsampling from 419 surgical patients at the open abdomen surgery unit at Karolinska University Hospital. Experiments with error mitigation are also tested. Results are compared with using the continuous blood pressure waveform data.Results: For a downsampling to once every 15 seconds, the relative bias was 27.0% for the number of hypotensive events, 30.0% for the cumulative hypotensive length, and 10.8% for the cumulative area under threshold. Error mitigation approaches often do not improve the errors.Conclusion: These results highlight the necessity of using high frequency data and that it is important to be aware of the errors when interpreting older studies involving quantification of hypotension and based on downsampled data.

Clinical and Translational Impact Statement: In our prospective study, we highlight accuracy problems when quantifying hypotension. In clinical settings, always use continuous blood pressure to quantify hypotension for individual patients to avoid accuracy errors.

## I. Introduction

Hypotension is the drop of blood pressure to a critical level and can lead to serious complications [1], [2], [3], [4], [5]. For instance, intraoperative hypotension (IOH) is associated with myocardial injury after noncardiac surgery (MINS) [5], [6], but also with acute kidney injury (AKI) and other complications [2]. Many of these complications are in turn associated with higher risk of death [7].

Both deeper and longer hypotension events are associated with higher risks of complications. However, how this association exactly looks like is partly unknown [8]. Hence, many studies have been conducted to shed light on this. In most of these studies, an important step is to quantify the amount of hypotension by using continuous intraoperative blood pressure measurements. Furthermore, in the future, this quantification may be used to decide whether the risk for post-operative complications in individual patients is so high that further follow-ups and treatments are required.

Several different definitions have been proposed [9], where many are based on mean arterial blood pressure (MAP) falling below either an absolute threshold, such as 65 mmHg, or a relative threshold based on a baseline value for the specific patient, such as a drop by 15% below baseline. It is also common to introduce a time limit, such as a period with MAP below the threshold for less than one minute is ignored. Alternatives and combinations also exist, including using systolic or diastolic pressure values instead of MAP [9]. Sometimes, the length and/or magnitude of the hypotension are weighted [8].

In modern patient monitors, the arterial blood pressure (ABP) is commonly captured using a catheter in the radial artery. A transducer converts the pressure to an electrical voltage, which is sampled and digitized by a sampling procedure [10] at 125 Hz or higher using 10-16 bit samples. Unfortunately, storing this high-frequency waveform data is still not standard practice, meaning that quantification of hypotension in past studies has seen many different approaches, including only saving parameter values once every 1 minute (or less) or even manually on paper. Reasons for this include lack of easy-to-use functionality to download waveform data or the large amount of data.

However, the extensive downsampling that these approaches infer, e.g., from 125 Hz to a single value once every minute, causes data loss and leads to estimation errors for key metrics, such as the metrics for hypotension. The non-linearity of common hypotension definitions aggravates this problem, such as the requirement of ignoring hypotension less than 1 minute.

In this article, we investigate what the reduction in sampling frequency has on accuracy for a few common definitions of hypotension. This is important so that we quantify hypotension in similar ways with good accuracy and can make the right conclusions in both scientific studies and for individual patients. It is known that severe downsampling reduces accuracy, but in this article, we also show how much bias downsampling introduces and that the bias is significant in most cases even with small amount of downsampling. Furthermore, we investigate mitigation approaches, such as bias compensation, where we can show that they cannot reduce the errors caused by downsampling, especially not for individual patients.

## II. Related Work

To the best of our knowledge, we are the first to study the measurement accuracy of common methods of quantifying hypotension. However, quantification of hypotension has been done in several previous articles, usually for the purpose of studying the effects of hypotension on patient outcomes. Besides using different definitions of hypotension [11], they also processed and quantified the blood pressure data in different ways. In the following, we give some examples:

• Bijker et al. [9] stored blood pressure (both invasive and non-invasive) values once every one minute, up to once every 5 minutes. 75 different hypotension definitions were tested.
• Walsh et al. [2] collected MAP values once every 1-2 minutes if using invasive or every 2-5 minutes if using non-invasive measurements. They calculated the cumulative length of hypotension using different MAP thresholds.
• Sun et al. [3] collected blood pressure values once every one minute. Cumulative length of hypotension based on MAP below an absolute threshold was used.
• van Waes et al. [4] used a system to calculate the average of MAP for every one minute. The used hypotension definition was lower than an absolute or relative threshold for one minute or longer. They calculated the number of hypotensive events, cumulative length of hypotension, and cumulative area under threshold (AUT).
• Salmasi et al. [5] recorded invasive pressures at 1-minute intervals, while non-invasive pressures were recorded at 1- to 5-min intervals.
• Gregory et al. [12] stated that MAP samples were collected at intervals up to a maximum of 5 minutes. The authors used the lowest recorded MAP value, cumulative length of hypotension, cumulative AUT, and time-weighted average (TWA) of MAP based on different MAP thresholds.
• Wesselink et al. [8] used the median ABP once every 1 minute. Hypotension was quantified as different types of weighted AUT.
• Both Mulder et al. [13] and Davies et al. [14] used averages of a period of 20 seconds to calculate hypotensive events longer than one minute.
• Saugel et al. [15] measured blood pressure once every 3 minutes using a non-invasive method, or once every one minute when using an invasive method. However, no minimal time for hypotension was imposed.
• Valadkhani et al. [16] used a sampling time of 15 seconds to calculate hypotension as well as tachycardia and used a minimum period time of 1 minute in both cases to define an event.

Some articles (e.g., [17], [18], [19]) do not report how blood pressure values were measured or analyzed. Furthermore, different articles also use different approaches to deal with noisy and missing data. The variation is just as big as the data collection and hypotension definitions mentioned above. Common methods are limit filters based on unrealistic values or too rapid changes. Missing values are sometimes filled in using carry-forward, interpolation, or excluded from the study.

We also note that the American Society of Anesthesiologists recommends to use non-invasive blood pressure (NIBP) reading at least once every 5 minutes [20].

## III. Methods and Procedures

Hypotension is often defined as MAP below a target threshold *T* for at least a period of time *L*. For IOH, $T = 65$ mmHg and $L = 1$ minute is one such definition [5]. In this article, we will use this definition but acknowledge the existence of alternative definitions and note that our results can be translated to other definitions, including definitions based on relative thresholds or systolic or diastolic blood pressures.

In addition, we define a *hypotensive period* as any period in which the MAP is continuously below the target threshold *T*. If such as period is longer than *L* minutes, we call it a *hypotensive event*.

To calculate the actual amount of hypotension, we use the ABP waveform data from the patient monitors during the period of interest, such as from the start of anesthesia to the point when the patient leaves the post-operative ward. The processing starts by finding the cardiac cycles for the recorded data of a patient *p*. This is done by a peak detection algorithm [21]. Let $T_{s}$ be the used sampling time of ABP and the diastolic peaks are detected at $\{ n_{p}[i] \}_{i \geq 0}$, which are indices in the sampled ABP signal. For a sampling frequency of 125 Hz, $T_{s} = 1/125 \;\mathrm {Hz} = 8$ ms.

The MAP within one cardiac cycle *i* with onset at $n_{p}[i]$ is the arithmetic mean and calculated as

\begin{align*} MAP_{p}[i] = \frac {1}{n_{p}[i+1] - n_{p}[i]}\sum _{m=n_{p}[i]}^{n_{p}[i+1] } ABP_{p}[m] \quad \mathrm {mmHg.} \tag {1}\end{align*}

where $ABP_{p}[m]$ are the instantaneous ABP values of patient *p* at sample time *m*.

We iterate over all cardiac cycles *i* to find periods of consecutive periods where $MAP_{p}[i] < T$. Each such period is indexed by *k* and its onset is the cardiac cycle $n_{p}[k]$ and has the length $\Lambda _{p}[k] = T_{s} (n_{p}[k+1] - n_{p}[k])$ seconds.

The second step in the process is to quantify this into a single scalar value. As mentioned in Section II, multiple methods have been proposed. In this work, we will use the following three key hypotension metrics for each patient *p*:

1) The total number of hypotensive events ($H_{p}$)
2) The cumulative length of all hypotensive events ($cLEN_{p}$)
3) The cumulative area under threshold of all hypotensive events ($cAUT_{p}$).

as defined here:

\begin{align*} H_{p} & = \sum _{k \geq 1} [ \Lambda _{p}[k] \geq L ] \tag {2}\\[0.1in] cLEN_{p} & = \sum _{k \geq 1} \Lambda _{p}[k] \cdot [ \Lambda _{p}[k] \geq L ] \tag {3}\\[0.1in] cAUT_{p} & = \sum _{k \geq 1} AUT_{p}[k] \cdot [ \Lambda _{p}[k] \geq L ] \tag {4}\end{align*}

where $[\cdot]$ is the Iverson bracket and:

\begin{equation*} AUT_{p}[k] = \frac {T_{s}}{60}\sum _{i = n_{p}[k]}^{n_{p}[k] + \Lambda _{p}[k]} T-MAP_{p}[i] \quad [\mathrm {mmHg \cdot ~min].}\end{equation*}

**FIGURE 1.**
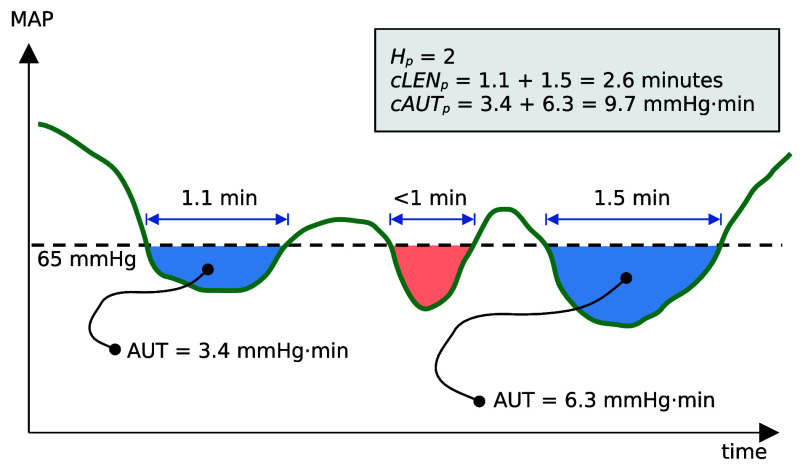
An example of a patient with three hypotensive periods and the corresponding hypotension metrics. Only two hypotensive periods (marked with blue) are longer than 1 minute and hence are hypotensive events.

The $cAUT_{p}$ is the sum of the AUT of all hypotensive events for patient *p* during the time frame of interest. TWA MAP is defined as $cAUT_{p}$ divided by the length of available ABP signal during the time frame of interest. Fig. 1 shows an example of the MAP values over time for a fictive patient *p*. In this example, there are two hypotensive events with a cumulative length of 2.6 minutes and a cumulative AUT of 9.7 mmHg$\cdot $min, since the second hypotensive period is too short (less than $L = 1$ minute) to be a hypotensive event.

### A. Preprocessing

In our study, the preprocessing started with high-frequency ABP waveform data from a patient monitor. Then, this procedure was followed:

1) Remove unrealistic ABP values, i.e., when too high (ABP > 250 mmHg) or too low (ABP < 15 mmHg). Also remove ABP values nearby, i.e., within ±2 seconds.
2) Remove too short ABP segments (less than 5 seconds)
3) Find both systolic and diastolic peaks based on a peak detection algorithm [21].
4) Remove cardiac cycles with unrealistic MAP values (MAP not between 35 and 150 mmHg), unrealistic cycle lengths (not between 10 and 300 BPM), or unrealistic pulse pressure (PP < 15 mmHg).
5) Remove too short ABP segments (less than 5 seconds) once again.
6) Skip patients with less than 90% ABP data from the surgery period.
7) Calculate the MAP for each cardiac cycle according to Eq. (1).
8) Fill small gaps (10 seconds or less) in the MAP data by linear interpolation.
9) Find all periods where all cardiac cycles have MAP values below the threshold *T*, i.e., all $n_{p}[k]$.
10) Calculate the hypotensive metrics according to Equations (2)–(4).

### B. Mean Arterial Pressure and Downsampling

The power spectral analysis of a typical ABP signal reveals that most of the energy is within the 0-15 Hz range, which means that a sampling frequency of 30 Hz can capture the wave form successfully. However, to detect the diastolic peaks and thereby the cardiac cycles, a lower sampling frequency is sufficient if using proper reconstruction methods. And to find the MAP of the cardiac cycles, an even lower sampling frequency is possible. However, for simplicity, it is easier to use the standard ABP sampling frequency of 125 Hz (or higher). After detecting the diastolic peaks, we can calculate the MAP for each cycle in a beat-to-beat manner, which constitutes a new derived signal. While it is possible to downsample the ABP signal, we will only focus on downsampling of MAP in this study since that is what previous studies have done.

Downsampling is the process with which we can reduce the amount of data. The MAP value will only change once per heartbeat. Hence, downsampling can remove redundant information if done correctly.

Standard temporal sampling and downsampling would mean that we continuously sample the MAP value with a regular rhythm, such as once per minute. The time between samples is called sampling time and denoted $T'_{s}$. Mathematically, we can express the temporal sampling process as follows:

\begin{align*} & MAP'_{p}[xT'_{s}] = MAP_{p}[i] \\ & \qquad \mathrm {with} \; \mathrm {the} \; i \; \mathrm {such} \; \mathrm {that} \; n_{p}[i] \leq xT'_{s} < n_{p}[i+1] \tag {5}\end{align*}

for all integers $x = \{ 0, 1, 2, \ldots, \lfloor \max _{i}(n_{p}[i]) / T'_{s} \rfloor \}$. This method is also known as impulse sampling. Alternatives include calculating the mean or median of all MAP values in each sampling period $T'_{s}$ instead [4], [8], such as:

\begin{align*} MAP^{mean}_{p}[xT'_{s}] & = mean_{i \in [A,B]}(MAP_{p}[i]) \tag {6}\\ MAP^{median}_{p}[xT'_{s}] & = median_{i \in [A,B]}(MAP_{p}[i]) \tag {7}\end{align*}

with $A, B$ such that:

\begin{align*} \begin{matrix} n_{p}[A]\leq & \qquad xT'_{s} \qquad {\,}{\,}< n_{p}[A+1] \\ n_{p}[B]\leq & \quad (x+1)T'_{s} \quad < n_{p}[B+1] \end{matrix}\end{align*}

Since the MAP value only can be defined per heart cycle at the highest resolution, beat-to-beat sampling of MAP is possible. Beat-to-beat sampling of MAP would mean that we find each heart cycle and calculate and store the MAP per cycle. Since the heart rate varies over time, the time between samples will vary in such a scheme. However, almost all previous papers have utilized temporal sampling with a fixed $T'_{s}$. In the remainder of this article, we will use $T'_{s} = 0$ to denote no downsampling, which also equals the beat-to-beat MAP signal. Using the beat-to-beat MAP signal constitutes the most accurate option and is therefore defined as the baseline.

### C. Effects of Downsampling

When downsampling the beat-to-beat MAP signal using a temporal approach, the estimated start is always delayed until the next sampling point. This delay can be modeled by a random variable, $\delta _{i}$. Similarly, the estimated end is delayed by a similar random variable, $\delta _{i+1}$. However, since the start and the end of a hypotensive period are random, they will both be uniformly distributed with the mean of half the sample time, i.e., $\delta _{x} \sim \mathcal {U}(0, T_{s})$. Furthermore, they will be independent variables, with the same expected value:

\begin{equation*} E[\delta _{i}] = E[\delta _{i+1}] = \frac {T_{s}}{2} \tag {8}\end{equation*}

This means that the estimate of the length of a hypotensive event is unbiased, which can be verified by measuring the collected data.

However, when we impose that hypotensive events must be longer than a time threshold *L*, then the downsampled estimator is no longer unbiased. The reason for this is not the threshold itself. Instead, downsampling may sometimes be tricked into thinking that two consecutive hypotensive periods are one long single period if they are close to each other. No sample may be taken in the short time between the two distinct periods, making the system believe it is one long period. This creates an overestimation and hence a positive bias if the two short periods are shorter than *L*, but the combined period is longer than *L*.

**FIGURE 2.**
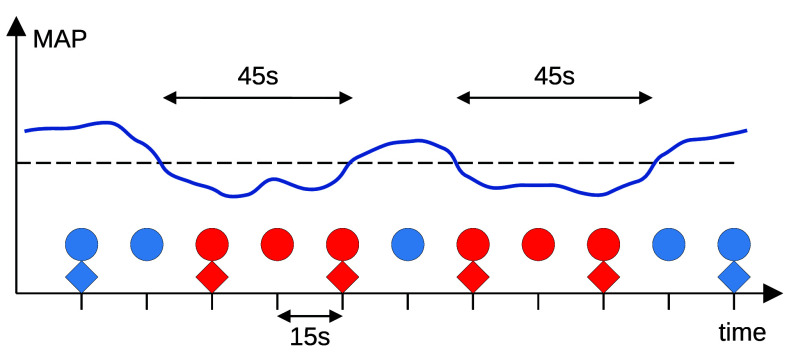
Example of the effect of downsampling on capturing hypotensive events.

Fig. 2 shows an example of two hypotensive periods separated by a short time of non-hypotension. If the sampling frequency is once every $T'_{s} = 15$ seconds (shown with circles), the two events will appear as two separate periods, both less than 45 seconds and therefore correctly ignored in the cumulative measures. However, if the sampling time is increased to $T'_{s} = 30$ seconds, then the two periods will appear as one long period as indicated by the continuous sequence of the four red diamonds. In this case, we will introduce a positive bias in the cumulative measurements.

Obviously, it is also possible to miss hypotensive events if the sampling time is too large. If a hypotensive event is 61 seconds, it is required that all four sampling points fall within the hypotensive event if $T'_{s} = 15$ seconds. In this case, the first sample must occur in the first second of the hypotensive event for all four samples to be within the hypotensive event.

It seems that it is more common that two smaller hypotension periods are merged by the downsampling process than missing short hypotensive events and this leads to overestimation. I.e., that the estimates of Eqs. (2)–(4) are larger: $\tilde {H}_{p} \gtrsim H_{p}$, $\widetilde {cLEN}_{p} \gtrsim cLEN_{p}$, and $\widetilde {cAUT}_{p} \gtrsim cAUT_{p}$. Therefore, we choose to investigate whether it is possible to rectify this by modifying Eqs. (2)–(4) by changing $[\Lambda _{p}[k] \geq L]$ into $[\Lambda _{p}[k] > L]$. This rectification means that for $T'_{s} = 15$, we need at least 5 sample points with $MAP < T$ since $T'_{s} \cdot 4 = 60 \ngtr L$ instead. We call this *Variant 2*, while the original version, specified in Eqs. (2)–(4) is called *Variant 1*.

### D. Aliasing and Sampling Artifacts

Aliasing can be a problem even though the MAP signal is not cyclical. There is a natural beat-to-beat MAP variation, where pressure change caused by breathing is a dominant factor. The pattern of a spontaneously breathing patient is very irregular and will cause less problems, but a mechanically ventilated patient with a regular breathing pattern can be. Typical mechanical ventilation has a cycle length of 4 to 5 seconds. Due to the Nyquist sampling criterion, we need a sampling rate of one sample every 2 seconds. Hence, aliasing can be a problem. However, the amplitude of the breathing effect is typically only a few mmHg. Nevertheless, when the MAP value is close to the threshold, breathing often creates several very short hypotensive periods as MAP follows the breathing. With downsampling, this effect can also be altered, leading to changes in the hypotension quantification.

### E. Patient Data

In this study, we used data collected from $n=457$ patients that underwent surgery at the open abdomen surgery unit at Karolinska, Sweden. In the supplement, we have data from an open data set VitalDB [22]. All data was used in accordance with the approval Dnr-2021-03073 and Dnr-2022-05955-02 of the Swedish Ethical Review Authority.

We only included the ABP data from the surgery and after preprocessing and removing noise, we had 419 patients with at least 90% ABP data, corresponding to 2,125.5 hours of surgery, 2,074.8 hours of ABP data, and 3,223 hypotensive events, which is the data set used in this study. Details about the excluded patients can be found in the supplement (Section S8).

### F. Simulation

Besides analyzing the ABP data from the patient data set, we also simulate patient data to better understand the underlying mechanisms, and experiment with a wider range of configurations. We choose to model the hypotension periods of a patient using a two-state stochastic process, where one state represents the hypotensive periods and the other state represents the periods in between. The simulator parameters are designed to match our patient data set. The details of the simulation design are presented in Section S1 of the supplemental material.

### G. Evaluation Measures

Since the true value differs a lot between patients, we most often use relative bias. However, we will also use the mean absolute error (MAE). The two error measures for the number of hypotensive events are defined as follows:

\begin{align*} \mathop {\mathrm {Bias}}\nolimits [\tilde {H}/H] & = \frac {\sum _{p \in P} \tilde {H}_{p}}{\sum _{p \in P} H_{p}} - 1 \tag {9}\\[0.1in] \mathrm {MAE}[\tilde {H}_{p}] & = \frac {1}{|P|}\sum _{p \in P} |\tilde {H}_{p} - H_{p}| \tag {10}\end{align*}

where *P* is the set of all patients ($|P| = 419$). The definitions for cumulative length and cumulative AUT are defined in the same fashion.

To calculate confidence intervals, we repeated each analysis 20 times with different start of sampling offsets. The 95% confidence interval was calculated and shown as error bars in all figures. For the simulations, we created 2,000 hypotensive periods as one patient case and repeated 50 times.

## IV. Results

In this section, we investigate the errors introduced by downsampling. Table 1 includes key metrics from the patient data used in most of these sections. Among all the included 419 patients, 390 patients (93.1%) had at least one hypotensive event.

**TABLE 1:** Patient Demographics and Key Hypotensive Measurements. $N=419$ Patients, SD is Standard Deviation

	Mean (SD) or N (%)	Min	Max
Age (years)	63.52 (12.41)	19	85
Sex, Male	179 (42.7%)		
Surgery length (hours)	5.07 (2.50)	2.0	14.85
Included ABP data (hours)	4.95 (2.42)	1.94	14.56
Mean MAP (mmHg)	74.42 (4.33)	64.41	96.56
$H_{p}$	7.69 (6.57)	0	38
$cLEN_{p}$ (minutes)	22.85 (23.78)	0.0	181.3
$cAUT_{p}$ (mmHg$\cdot $min)	117.7 (130.4)	0.0	711.2
TWA MAP (mmHg)	0.42 (0.46)	0.0	3.24

### A. Downsampling Results

**FIGURE 3.**
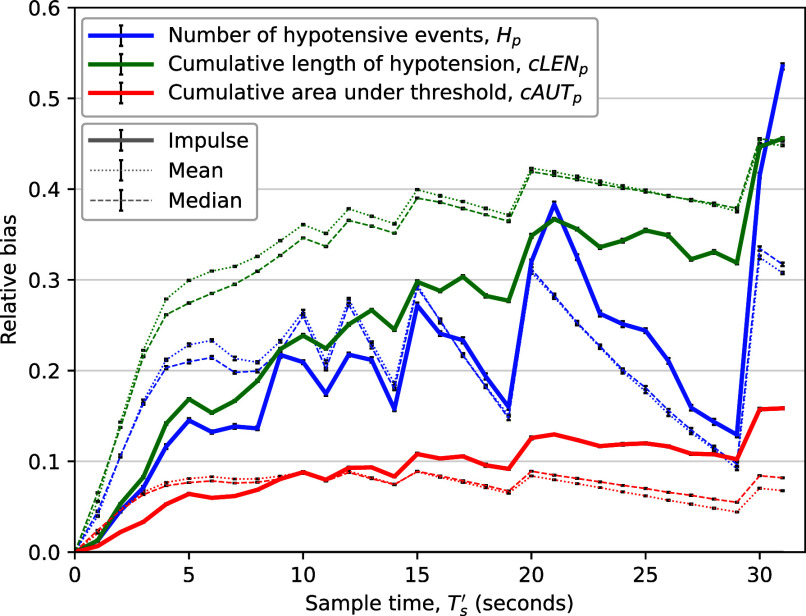
Relative bias (Eq. 9) for different amount of downsampling on number of hypotensive events (blue), and cumulative length of hypotensive events (green), and cumulative area under threshold (red). Only variant 1. Thin dotted and dashed lines indicate the results of the alternative sampling methods using the mean or median over the entire sample time.

**FIGURE 4.**
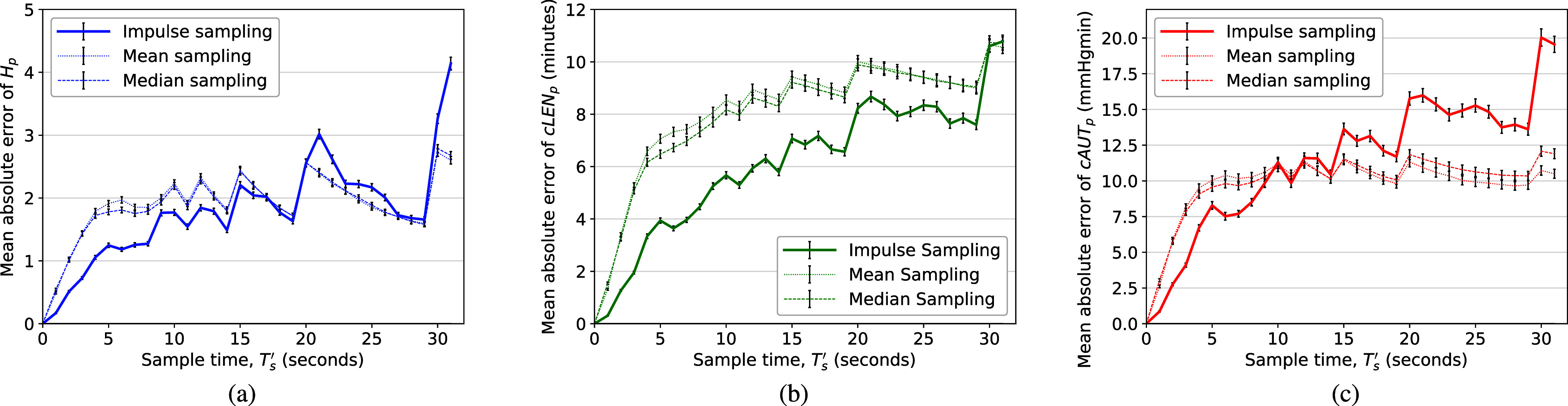
Mean absolute errors (MAE) (Eq. 10) for different amount of downsampling on number of hypotensive events (blue), and cumulative length of hypotensive events (green), and cumulative area under threshold (red). Only variant 1. Thin dotted and dashed lines indicate the results of the alternative sampling methods using the mean or median over the entire sample time. (a) Number of hypotensive events, $H_{p}$. (b) Cumulative length of hypotensive events, $cLEN_{p}$. (c) Cumulative area under threshold, $cAUT_{p}$.

Fig. 3 shows how different amount of downsampling affects the quantization of hypotension according to all three metrics. The MAE of all metrics are shown in Fig. 4. The value $T'_{s}=0$ is with no downsampling and is used as the reference value. The graph shows how the bias increases as the amount of downsampling increases for all three metrics. The cumulative length $cLEN_{p}$ is most affected and the number of hypotensive events $H_{p}$ fluctuates the most with different $T'_{s}$. As an example, if we downsample to once every $T'_{s} = 15$ seconds, we will over-estimate the number of events by 27.0%, the hypotension length by 30.0%, and the area under threshold by 10.8%. Expressed as MAE, this corresponds to 2.2 events, 7.1 minutes, and 13.6 mmHg$\cdot $min, respectively.

The reason for the sharp fluctuations between the different amount of downsampling can be explained by the interplay between the sampling process (i.e., $T'_{s}$) and *L*, the minimum length of the hypotensive event. It can clearly be seen from the figure that a sample time $T'_{s}$ that is a proper fraction of the length, i.e., $T'_{s} = \{1, 2, 3, 4, 5, 6, 10, 12, 15, 20, 30, \ldots \}$ often causes a larger bias. Hence, it is better to choose a sampling time of $T'_{s} = 11$, 14, 19, or 29 seconds if possible. In the supplement, we show what happens for different *L* and as expected, the bias mainly shifts between the $T'_{s}$ values.

In Fig. 3 and Fig. 4, we also show results for the different sampling methods as defined in Eq. (5) and Eqs. (6, 7). The colors of the curves denote different metrics, while the line types distinguish between the different sampling methods. For the cumulative hypotension length, mean and median sampling increases the relative bias even further. For the number of hypotension events and the cumulative AUT, the relative bias also increases, but only until a sample time of about $T'_{s} = 10$ seconds. After that the relative bias decreases for both metrics. However, the relative bias still remains large in most cases.

### B. Bias Compensation

From Fig. 3, we can see that all curves have a positive bias. Due to this, we may consider a bias compensation. A compensation can be adding or multiplying a constant to the original estimate in order or obtain a better estimate. However, we need to make sure we select the best possible constant and from Fig. 3 we can see that this constant depends on $T'_{s}$, variant, and the metric of interest, but all these can be determined beforehand. Unfortunately, the constant also depends on the frequency of hypotensive periods.

**FIGURE 5.**
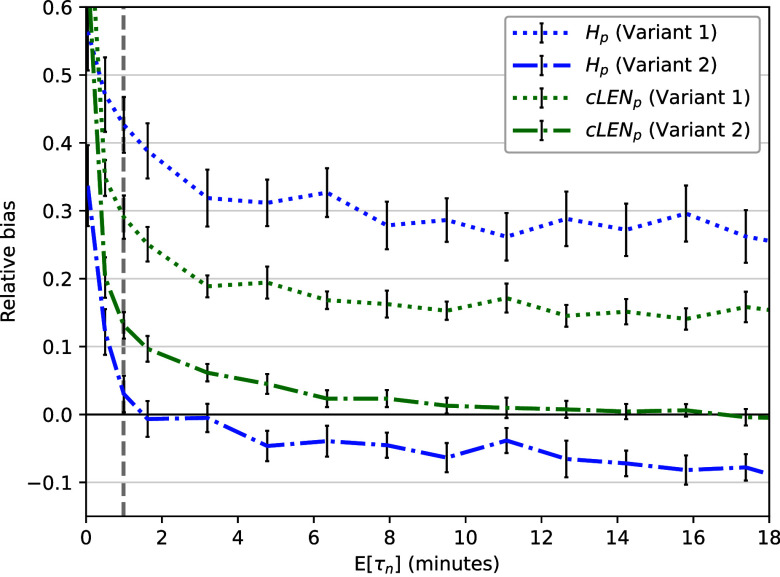
Sensitivity with respect to patients with different number of hypotensive periods per time unit. Showing Relative bias (Eq. 9).

To simulate patients with different amount of hypotension, we modified the time between hypotensive periods in the simulator. In Fig. 5, we show the relative bias of the number of hypotensive events and cumulative length. In this example, we used $T'_{s} = 15$ seconds. On the x-axis we place the mean value of this distribution ($E[\tau _{n}]$), where $\tau _{n}$ is the time between two hypotensive periods. The dashed vertical line is the original case based on the patient data. A larger value could represent a patient less likely to experience hypotension, while a smaller value represents patients with more hypotension. The MAE results are available in the supplement (Fig. S8). We can see that the relative bias is radically shifting and that different patients must have different amount of compensation, which makes this approach difficult to use in practice.

Another way to investigate this is to look at the bias, error, and variance when compensating. In Table 2, we consider the errors for all evaluation metrics and all measures from the patient data set for Variant 1 with sampling time $T'_{s} = 15$ seconds for both with and without bias compensation. Results from Variant 2 are available in the supplement (Table S2) but exhibits a similar behavior.

**TABLE 2:** The Effect of Bias Compensation, Variant 1

$H_{p}$	$\gamma =1$		$\gamma =1/1.4$	
$\mathop {\mathrm {Bias}}\nolimits [\gamma \tilde {H}/H]$	1.99	(2.68)	−0.683	(2.144)
$\mathop {\mathrm {Bias}}\nolimits _{p}[\gamma \tilde {H}_{p}/H_{p}]$	0.370	(0.568)	−0.028	(0.420)
MAE	2.19	(2.52)	1.57	(1.61)
$cLEN_{p}$	$\gamma =1$		$\gamma =1/1.4$	
$\mathop {\mathrm {Bias}}\nolimits [\gamma \tilde {LEN}/LEN]$	7.04	(7.50)	−1.68	(5.89)
$\mathop {\mathrm {Bias}}\nolimits _{p}[\gamma \tilde {cLEN}_{p}/cLEN_{p}]$	0.422	(0.569)	−0.017	(0.415)
MAE	7.06	(7.49)	3.86	(4.76)
$cAUT_{p}$	$\gamma =1$		$\gamma =1/1.2$	
$\mathop {\mathrm {Bias}}\nolimits [\gamma \tilde {cAUT}/cAUT]$	13.2	(19.3)	−9.29	(21.33)
$\mathop {\mathrm {Bias}}\nolimits _{p}[\gamma \tilde {cAUT}_{p}/cAUT_{p}]$	0.174	(0.304)	−0.021	(0.253
MAE	13.7	(19.0)	14.5	(18.2)

The effect of bias compensation on different bias and error metrics. Sampling time is set to $T_s^{\prime}=15$ seconds. The bias compensation is done by multiplying a factor $\gamma$ with the original estimate. Values in parentheses are the standard deviation.

To compensate, we use a constant factor that is multiplied with the original estimate. Since this is applied on a patient by patient basis, we introduce a second slightly different definition of the relative bias as follows:

\begin{equation*} \mathop {\mathrm {Bias}}\nolimits _{p}[\tilde {H}_{p}/H_{p}] \; = \; \frac {1}{|P'|}\sum _{p \in P'} (\tilde {H}_{p}/H_{p} - 1) \tag {11}\end{equation*}

where $P' = \{ p \in P: H_{p} > 0\}$ is the set of all patients with at least one hypotensive event ($|P'| = 390$). The definitions for cumulative length and cumulative AUT are defined in the same fashion. This definition, which first calculates the bias for each patient *p* and then calculates the mean of all those biases, is used to determine the size of the compensation.

The amount of compensation, which we denote $\gamma $, is derived from the actual relative bias (Eq. 11) from the patient data set. From the no bias compensation ($\gamma = 1$) results of Table 2, we see that the relative bias (Eq. 11) is 37% for variant 1 and number of hypotensive events, which mean that we use $\gamma = 1/1.4$ for the number of hypotension events with Variant 1. For cumulative hypotensive length, a similar $\gamma $ can be used. However, for cumulative AUT, we see an overestimate of around 17%, which means $\gamma = 1/1.2$ is better here.

On the population level, we can see that the relative bias (Eq. 11) is reduced to close to zero (−0.028, −0.017, and −0.021 respectively) due to the bias compensation, which is expected. However, the same cannot be said for the other metrics. The original relative bias (Eq. 9) is shifted to negative, meaning an underestimation instead. The MAE does not always reduce and is still not close to zero. The same, we see for the standard deviation as well. Nevertheless, it can be concluded that compensation on the individual patient level does not lead to a better accuracy.

## V. Discussion

To be able to size the amount of hypotension that a patient is exposed to has many important usages. We have seen numerous studies trying to link the amount of hypotension with several post-surgical complications [1], [2], [3], [4], [5]. A more recent development is using machine learning (ML) to either predict the near onset of hypotension [13], [14] or post-surgical complications [16], [18]. To compare between studies, or to use different datasets for training ML models, we first need to use the same definition of hypotension [9]. However, it is also important that the quantification of hypotension is similar or at least that we understand the differences.

Our research clearly demonstrates that different ways of calculating hypotension, even if we use the same definition of hypotension, can yield very different results. As illustrated by Fig. 3, the errors depend on the amount of downsampling, the sampling method used, and often becomes significantly large. Already at $T_{s} > 5$ seconds, the errors are significant with a typical overestimation of 15% or more.

The overestimation is more severe for the number of hypotensive events and the cumulative length than for the cumulative AUT. Hence, cumulative AUT is a more robust metric. This is due to that shorter hypotensive periods tend to be shallower and therefore contribute less to the cumulative AUT metric. Hence, a short missing hypotensive period has limited effect on the cumulative AUT metric.

The reason for overestimation when using downsampling is usually due to accidental period merging. If the time between two consecutive periods is similar to or smaller than the sampling time, it may be interpreted as one single period after downsampling as illustrated by Fig. 2. The problem is the minimum 1-minute rule. When dealing with downsampled data, we should avoid this rule if possible, or avoid using the number of hypotensive events altogether and instead rely on the cumulative AUT, perhaps using weighting (e.g., [12]).

**FIGURE 6.**
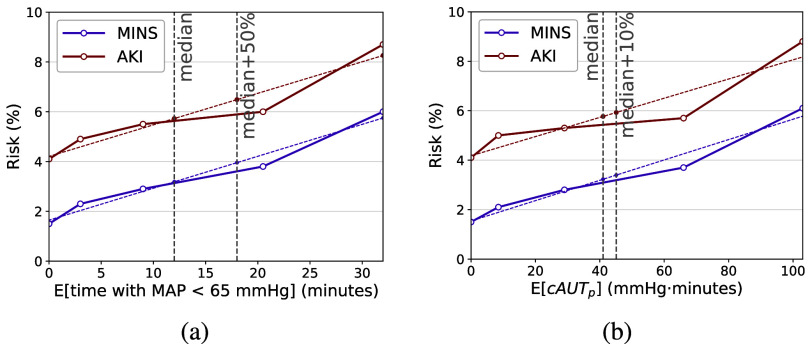
Risk of MINS and AKI given different amount of IOH. Based on (a) $cLEN_{p}$, (b) $cAUT_{p}$.

If the errors are consistent, they could be tolerated as long as the same method is used for model development and model use. The same holds for machine learning-based models. When comparing two different studies, it is difficult to compare the results if they use different methods. To exemplify these problems in a clinical context, assume a positive overestimation of + 50% when using $cLEN_{p}$ and + 10% when using $cAUT_{p}$, which are not uncommon in some of our results. It is difficult to estimate exactly what such an error would mean in terms of error in a clinical risk stratification step. Using the risk prediction results of Salmasi et al. [5], we can estimate the errors based on their presented data of how different errors in time with IOH ($cLEN_{p}$) and AUC ($cAUT_{p}$) correspond to the risk of MINS and AKI. Using a linear fit and assuming a patient with the median amount of IOH, we can estimate the relative error in the risk stratification to be about + 24% for MINS and + 13% for AKI when using $cLENp$ and about + 5% for MINS and + 3% for AKI when using $cAUT_{p}$, as shown in Fig. 6. The details of this evaluation is in the supplement, Section S5.

So far, we have presented a single-center study. However, we also did large parts of this study with the openly available VitalDB data set [22] as well. The results are shown in detail in the supplement, but are in line with the presented results, with only smaller variations.

Most studies use impulse sampling, while a few used mean or median sampling when downsampling. As we can see in Fig. 3 and Fig. 4, it usually does not reduce the error. Hence, we cannot recommend such methods.

Bias compensation, or switching to Variant 2, can be applied to reduce errors as shown in Table 2 and the same table for Variant 2 in the supplement. However, the best bias compensation ($\gamma $) may vary from data set to data set, between metrics and patients. Since the optimal $\gamma $ of Table 2 may not be optimal for another data set, these approaches have limited applicability. Furthermore, the variance still remains large. For most patients, it is an overestimation, but for some it is an underestimation. Hence, a fixed bias compensation will increase the error for some patients. So clinically, this approach should not be used for individual patient inference. However, for approximate population-based retrospective studies, bias compensation can be considered, but still understanding that it does not fully eradicate the errors.

In the past, it has been difficult to extract high-frequency data from patient monitors, but that is no longer the case. Non-invasive methods that provide continuous blood pressure values have been available for several decades [23]. All major manufacturers of modern patient monitors offer support for exporting high-frequency data, including waveform data. Given the large accuracy errors that we have demonstrated, and all the problems with the rectification approaches, we can only recommend to use continuous high-frequency blood pressure data today. This is also in line with existing literature that highlights the clinicians need for continuous monitoring [17]. Today’s barriers to use high-frequency data are a lack of efficient ways to collect, store, and handle such data and the large amount of data a hospital generates. Compression can provide a solution [24]. Another solution is to store only the beat-to-beat MAP values, which also reduces the storage requirements significantly.

## VI. Conclusion

The monitoring of hypotension is critical during surgical procedures. Given the established correlation between hypotension and post-operative complications, it is imperative to quantify hypotension. In this work, we have demonstrated that quantifying hypotension using parameter data at low frequencies introduces very large measurement inaccuracies. Especially the number of hypotensive events and the cumulative length are sensitive to overestimating when using low-frequency sampling. These findings underscore the necessity for continuous monitoring of blood pressure using high-frequency waveform data instead. When interpreting older studies or using older data sets, we can use bias compensation, but only at population level and never for individual patients.

## Supplementary Materials

Supplementary Materials
