# Supplementary material for: Accuracy of Quantifying Hypotension During Surgery Using Physiological Sensor Data
Source: IEEE J Transl Eng Health Med. 2026 May 29;14:265–73. doi: 10.1109/JTEHM.2026.3698197 (PMC13278748; doi:10.1109/JTEHM.2026.3698197)
Supplement: Supplementary Materials [file supp1-3698197.pdf]

Accuracy of Quantifying Hypotension During Surgery Using Physiological Sensor Data: Supplemental Information

Martin Jacobsson, Arman Valadkhani, Greg Winski, Max Bell

May 26, 2026

S1 Simulation

Besides analyzing the arterial blood pressure (ABP) data directly from the patient data, we also simulated patient data. In this section, we provide a more detailed description of the developed simulation model.

The hypotension periods of a patient is modeled using a two-state stochastic process, where one state represents the hypotensive periods and the other state represents the periods in between. The process stays in the two states for a length of time (sojourns time) modelled by two different continuous distributions. All period lengths are independent variables. This process is similar to a continuous-time Markov chain with two states, but not with exponentially distributed sojourn times.

We used the patient data to identify the sojourn time distributions and to tune the simulation parameters. We calculated the lengths of all hypotensive periods from all patients as well as all the inter-spacing times between hypotensive periods, in total 102,492 periods of each type. We created histograms for both period types and tried to find the distributions that best describe them. The distributions are not easily modeled. We tried several standard distributions, including Lognormal, Gamma, and Weibull distributions with different ways to correctly parameterize them. We used fitting using methods of moments as well as goodness of fit, probability plots, and visual inspection of the probability density function (PDF) and data histograms. In the end, we selected Weibull for both hypotensive and in-between periods.

Let τ_h be the length of a hypotensive period and τ_n be the length of an

in-between period. Then we model the periods as follows:

$$\begin{aligned} \tau_h &\sim 0.5 + \text{Weibull}(\lambda_h, k_h) \\ \tau_n &\sim 0.5 + \text{Weibull}(\lambda_n, k_n) \end{aligned} \tag{1}$$

where λ is a scaling parameter and 0.5 models the smallest possible period length (determined by a very small heart cycle). In our simulation, $k_h = 0.35$, $k_n = 0.36$, $\lambda_h = 1.7480$, and $\lambda_n = 12.961$. This gave us the same mean as the patient data, but a smaller median.

Figure S1 shows the final fit where we used the method of moments to find the distribution parameters that generates the distribution with identical mean to the patient data. The mean and median values of the patient data and the fitted distributions are shown in Table S1.

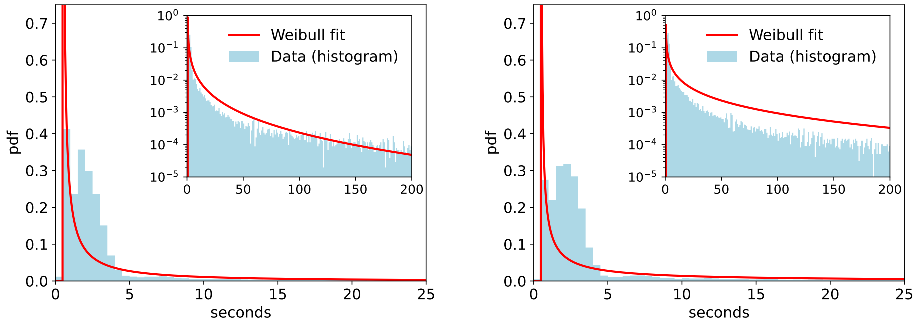

(a) (b)

Figure S1: Probability distribution function (PDF) of the chosen Weibull distributions and histogram of hypotensive periods. The blue is the histogram from the complete data set. The red curve is the PDF of the fitted Weibull distribution. (a) Hypotension period length (τ_h), (b) In-between times (τ_n)

In Figure S1, we can see that the fit is good for large values, but there is a mismatch between the patient data and the Weibull distribution for periods smaller than 5 seconds. It is likely three different processes acting in the data. One is the patient hemodynamics, which dominates in the larger values and it tends to Weibull. This is the one we are interested in here. The second process is breathing. This affects the period lengths when a patient has a MAP close to the threshold T. We can see a small peak at around 2.5 seconds in both histograms, which corresponds very well with

Table S1: Mean and median of the simulation and patient data

	$E[\tau_h]$	median(τ_h)	$E[\tau_n]$	median(τ_n)
Patient Data	9.29	2.05	59.7	2.48
Simulation	9.29	1.11	59.7	5.18

All values in seconds.

the common mechanical ventilation rate of 12-14 BPM (a cycle length of 2.5+2.5 seconds). The final process is other types of noise, which will also affect period lengths when the patient has a MAP around the threshold, but in a more random way. The corresponding probability plots are shown in Fig. S2.

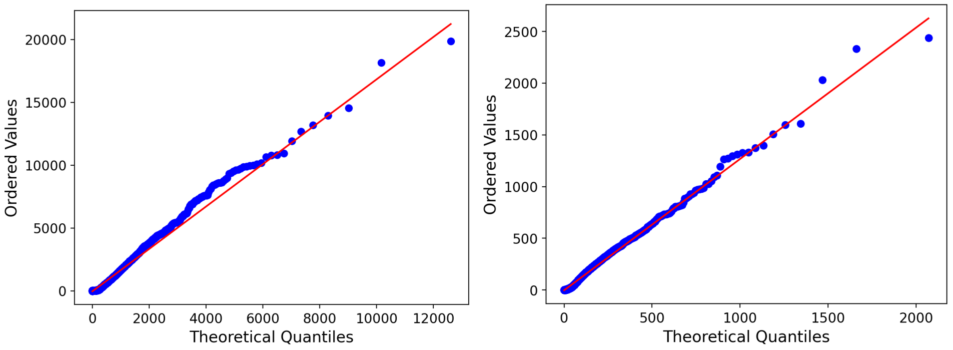

(a) (b)

Figure S2: Probability plots for the chosen Weibull distributions. (a) In-between times, (b) Hypotension period length

S2 Additional Analysis

Figure S3 illustrates the mean absolute percentage error (MAPE) for different amounts of downsampling for all three metrics and all three sampling types according to the following definition of MAPE:

$$\mathrm{MAPE}[\tilde{H}_p] = \frac{100\%}{|P'|} \sum_{p \in P'} \frac{|\tilde{H}_p - H_p|}{H_p}$$

where $P' = \{p \in P : H_p > 0\}$ is the set of all patients with at least one hypotensive event ($|P'| = 390$). The definitions for cumulative length and cumulative AUT are defined in the same fashion. Note that the results of Figure S3 do not include patients without hypotension ($N = 390$).

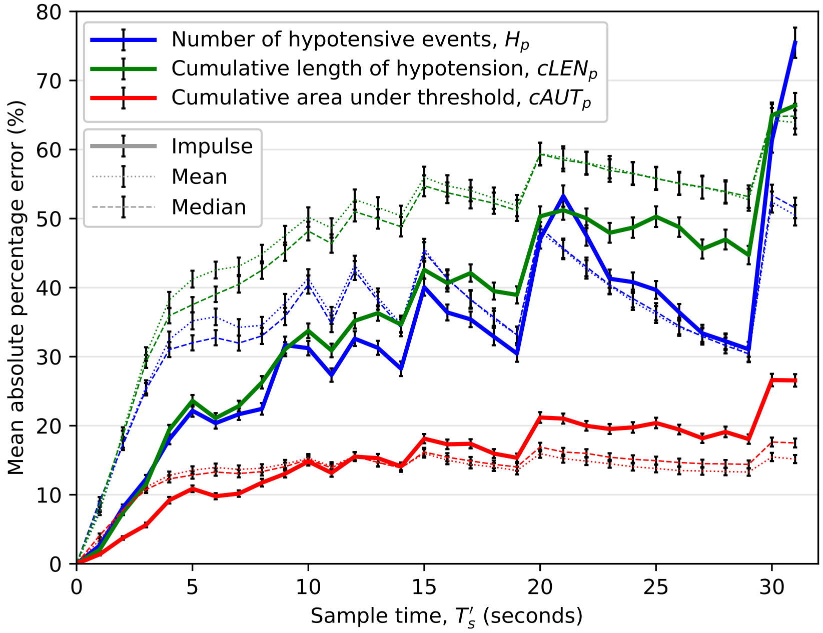

Figure S3: Mean absolute percentage error (MAPE) for different amount of downsampling on all metrics and all three sampling types.

Time-weighted average (TWA) of MAP is defined as the $cAUT$ divided by the length of the available ABP data during the time frame of interest. This metric is similar to $cAUT$, but removes the surgery length aspects. The relative bias (Eq. 8) and MAE are shown in Figure S4. From the figure, we can see similar results as $cAUT$; the relative bias is similar, while the MAE is different only due to the different scales.

S3 Additional Simulation Results

The graphs in Figure S5 show simulation and patient data results for both variant 1 and variant 2, where we change the time condition from a non-strict

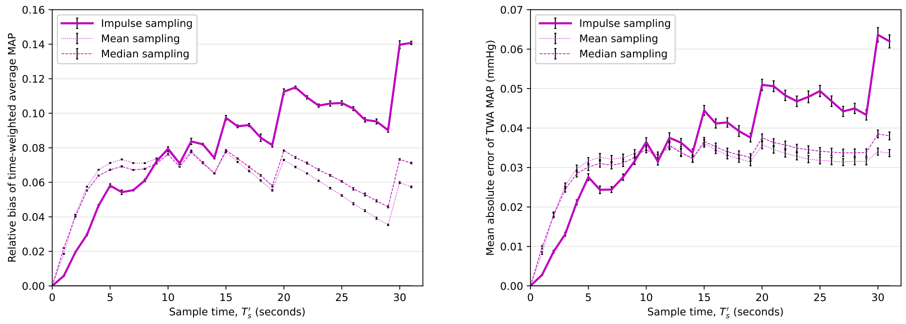

(a) (b)

Figure S4: Error measures for time-weighted average (TWA) of MAP using different amount of downsampling. (a) Relative bias (Eq. 8) (b) Mean absolute error (MAE)

comparison ($\geq$) to a strict comparison ($>$).

Figure S6 shows MAPE results from simulation and Figure S7 shows the MAE results. However, we do not model the length of surgery, so we cannot compare these results with the patient data.

All the results are similar to the Variant 1 results, but with the peaks and valleys shifted slightly. This approach can be beneficial when the minimum hypotension length L and the sampling period T_s' lead to a peak in the error metrics, such as for $L = 1$ minute and $T_s' = 15$ seconds. For other L and T_s' combinations, the error can increase.

S4 Bias compensation, Variant 2

Figure S8 shows the MAE for the bias compensation simulation. Table S2 shows the variant 2 of the bias compensation.

S5 Bias implication on risk stratification

In this section, we will try to estimate what the error in IOH estimation could mean clinically. Specifically, we will look at the risk stratification of PMI (or MINS) and AKI. We base our study on the work by Salmasi et al. [1], which includes data on what different amount of IOH would mean in terms of risk of MINS and AKI. We acknowledge, but ignore, the fact that

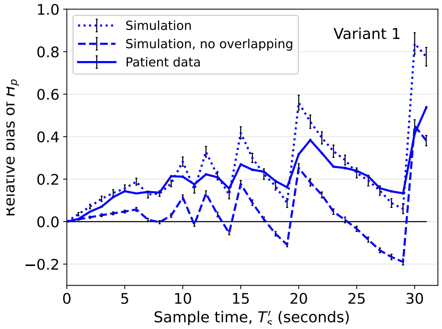

(a)

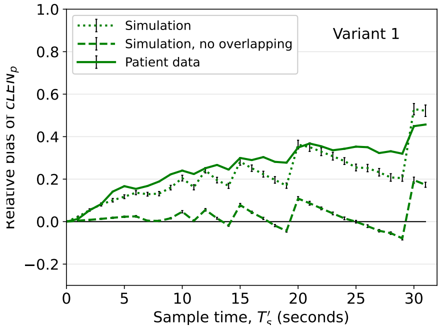

(b)

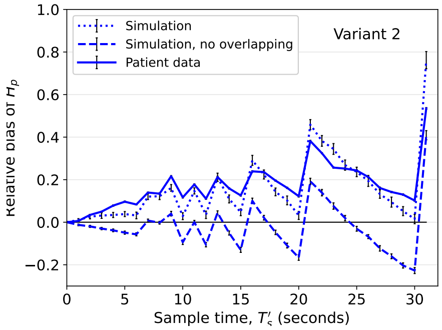

(c)

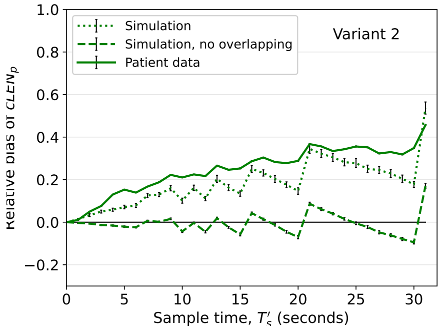

(d)

Figure S5: Relative bias (Eq. 8) for different amount of downsampling on number of hypotensive events and cumulative length of hypotensive events using patient data and simulation. (a) Number of hypotensive events, H_p, variant 1 (b) Cumulative length of hypotensive events, $cLEN_p$, variant 1 (c) Number of hypotensive events, H_p, variant 2 (d) Cumulative length of hypotensive events, $cLEN_p$, variant 2

the work of Salmasi et al. already has a positive bias due to their method used to estimate the amount IOH.

In Table 4 and 5 of Salmasi et al. [1], we find that the patients have been stratified in five categories based on how much IOH they were exposed to according to various definitions. In this analysis, we will focus on time with MAP<65 mmHg (similar to $cLEN_p$) and area under curve (AUC), which we understand as similar to $cAUT_p$.

In the presented data [1], we do not know the average amount of IOH for

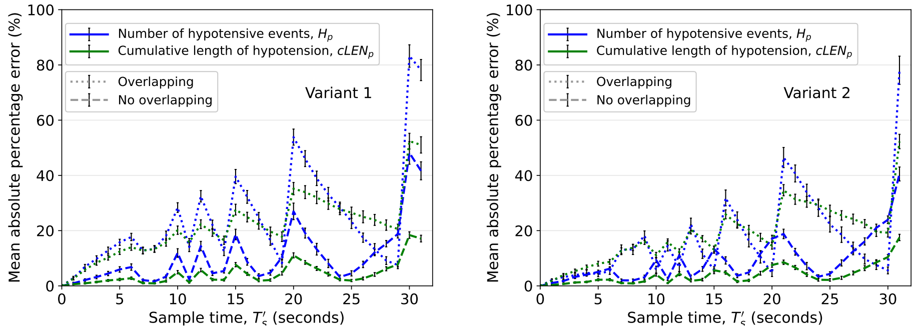

(a) (b)

Figure S6: Mean absolute percentage error (MAPE) for different amount of downsampling on number of hypotensive events and cumulative length of hypotensive events using simulation. (a) Variant 1 (b) Variant 2

the five categories. Instead we estimate the average by means of interpolation and extrapolation. When a range is presented, we use the average value of the upper and lower limits (i.e., for the category of 1–16 mmHg·min of AUC, the average is assumed to be 8.5 mmHg·min). For the final category, we use extrapolation of the last limits (i.e., for the category of AUC > 90 mmHg·min, we use $90 + 90 - 41 = 103$ mmHg·min)

We scatter plot the results, but since we only have five categories and very few data points, we assume a linear relationships between the amount of time in IOH (i.e., $cLEN_p$) or AUC (i.e., $cAUT_p$) and the risk of MINS/AKI. Fig. 6 in the article shows the scatter plot and the linear fits (dashed lines) for all four combinations. As seen in figure, a possible overestimation (i.e., bias) in calculating the $cLEN_p$ can be up to about 50% according to our results. The overestimation is lower for $cAUT_p$, but can still account for as much as +10%. In a fictive example, assume that these overestimations occur. Furthermore, assume a patient with median amount of IOH, which we find from the category limits of Salmasi et al. [1] (i.e., 12 minutes and 41 mmHg·min). These cases has been indicated with vertical lines in Fig. 6 of the main article. The overestimation of the risk for PMI and AKI are the vertical differences between the small solid dots on the linear fits. In Table S3, we include both the relative error and the absolute error to all risk predictions. There are other studies [2, 3] with similar data, and they show similar results.

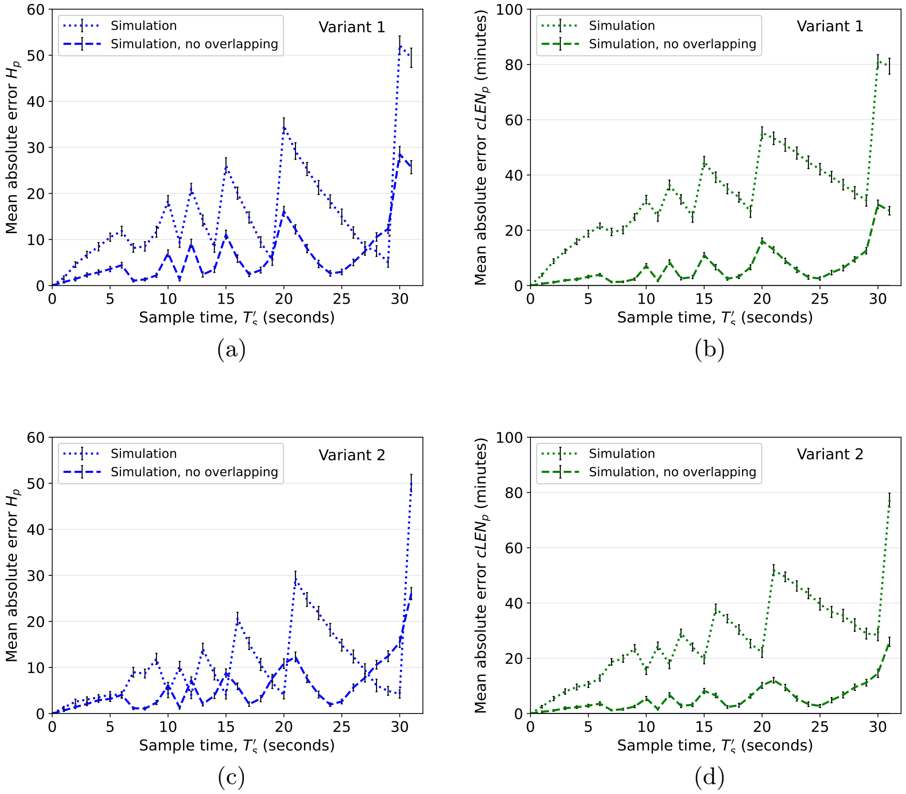

(a) (b) (c) (d)

Figure S7: Mean absolute error (MAE) for different amount of downsampling on number of hypotensive events and cumulative length of hypotensive events using simulation. (a) Number of hypotensive events, H_p, Variant 1. (b) Cumulative length of hypotensive events, $cLEN_p$, Variant 1. (c) Number of hypotensive events, H_p, Variant 2. (d) Cumulative length of hypotensive events, $cLEN_p$, Variant 2.

S6 Varying the minimum length of hypotension

It is common to exclude hypotensive periods less than one minute long as such periods have negligible impacts on patients. As a consequence, we used $L = 60$ seconds throughout the article. The implication of different L is shown in Fig. S9.

From Fig. S9, we can see that the effect is very similar irrespective of the chosen L. However, the peaks and valleys are shifted along the sample

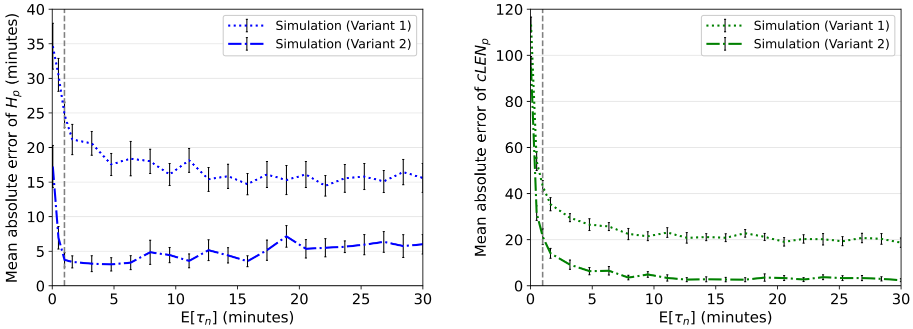

(a) (b)

Figure S8: Sensitivity with respect to patients with different amount of hypotensive periods per time unit. Showing mean absolute error (MAE). (a) Number of hypotensive events, H_p (b) Cumulative length of hypotensive events, $cLEN_p$

time T_s'. As explained in the article, the peaks are created mainly when the sampling time is a proper fraction of L.

S7 Cross-Clinical Validation

The data in the article is based on a data set collected at the Karolinska University Hospital, Stockholm, Sweden. In this section, we try another openly available data set, namely the VitalDB dataset [4], which contains vital signs, including ABP, from 6,388 surgeries at the Seoul National University Hospital, Seoul, Republic of Korea. Furthermore, this data set contains different types of surgeries (general, thoracic, urologic, and gynecologic).

To conduct a similar validation as we did with the Karolinska, we cannot include all patients from VitalDB. We selected patients according to the following criteria: Only adult patients ($\geq$ 18 years old) with general anesthesia, ASA $\leq$ 4, only surgeries that lasted at least 2 hours, only patients having a left or right radial arterial line, and with the availability of the ABP signal. It was 2,119 surgeries that fulfilled these criteria. Furthermore, we only included the ABP signal from the start of surgery to the stop of surgery and excluded cases if more than 10% of the ABP signal was missing during the surgery. This gave us $N = 1,964$ patients from the VitalDB data set. Finally, we downsampled from 500 Hz to 125 Hz using an FFT-based

Table S2: The effect of bias compensation, variant 2

H_p	$\gamma = 1$		$\gamma = 1/1.2$	
Bias$[\gamma\tilde{H}/H]$	0.918	(2.04)	-0.510	(1.897)
Bias$_p[\gamma\tilde{H}_p/H_p]$	0.194	(0.462)	-0.0091	(0.3729)
MAE	1.41	(1.74)	1.34	(1.43)
$cLEN_p$	$\gamma = 1$		$\gamma = 1/1.4$	
Bias$[\gamma c\tilde{LEN}/cLEN]$	5.97	(6.84)	-2.45	(5.89)
Bias$_p[\gamma c\tilde{LEN}_p/cLEN_p]$	0.343	(0.510)	-0.0383	(0.3750)
MAE	6.03	(6.79)	4.06	(4.91)
$cAUT_p$	$\gamma = 1$		$\gamma = 1/1.1$	
Bias$[\gamma c\tilde{AUT}/cAUT]$	10.1	(16.6)	-1.82	(15.40)
Bias$_p[\gamma c\tilde{AUT}_p/cAUT_p]$	0.127	(0.270)	-0.027	(0.258)
MAE	11.2	(15.9)	9.53	(12.22)

The effect of bias compensation on different bias and error metrics. The bias compensation is done by multiplying a factor γ with the original estimate. Values in parentheses are the variance. For $T'_s = 15$ seconds.

Table S3: Errors in risk prediction with overestimation

	$cLEN_p$ + 50%		$cAUT_p$ + 10%	
Risk	relative error	absolute error	relative error	absolute error
MINS	24%	0.77%	5.2%	0.17%
AKI	13%	0.76%	2.8%	0.16%

method (SciPy resample) to be able to run it through the same processing pipeline as the Karolinska data set. The patient characteristics of the included patients are given in Table S4.

The first test using the VitalDB data set is to see how the errors occur

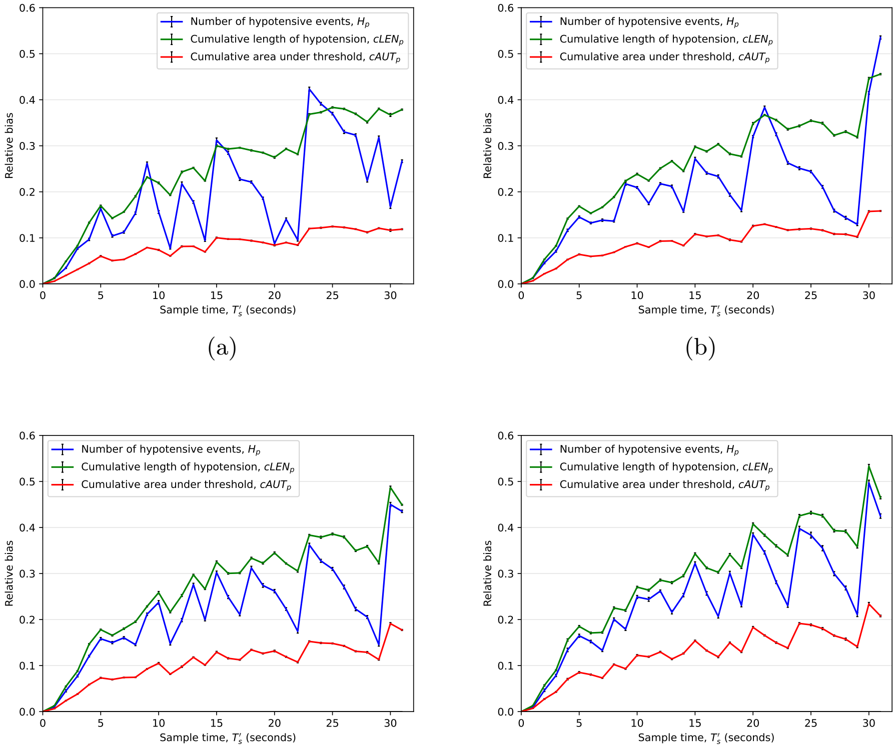

(a) (b) (c) (d)

Figure S9: Relative bias (Eq. 8) for different amount of downsampling on number of hypotensive events and cumulative length of hypotensive events. Variant 1 and Impulse sampling method. (a) $L = 45$ seconds. (b) $L = 1$ minute. (c) $L = 1.5$ minutes. (d) $L = 2$ minutes.

under different amount of downsampling. The relative bias (Eq. 8) and MAPE of the error metrics are shown in Figure S10. As before, only patients with at least one hypotensive are included ($N = 1,088$) in the MAPE results. As can be seen, there is great similarity with the results from the Karolinska data set, which also includes the MAE results not shown here. The peaks and valleys occur at the same amount of downsampling. However, the relative bias (Eq. 8) is somewhat lower, while the MAPE is more similar. This can be explained by the larger amount of patients without hypotension (45% in VitalDB vs. 7% in Karolinska).

The second step is to do the simulation modeling using the VitalDB data

Table S4: Patient Demographics and Key Hypotensive Measurements for the VitalDB data set. $N = 1,964$ patients, SD is standard deviation.

	Mean (SD) or N (%)	Min	Max
Age (years)	59.5 (13.85)	18.0	92.0
Sex, Male	1197 (60.9%)		
Surgery length (hours)	3.72 (1.49)	2.0	15.67
Included ABP data (hours)	3.62 (1.44)	1.81	15.21
Mean MAP (mmHg)	83.4 (9.5)	56.2	120.6
H_p	3.54 (6.41)	0	72
$cLEN_p$ (minutes)	13.12 (28.21)	0.0	308.4
$cAUT_p$ (mmHg·min)	82.85 (220.4)	0.0	3346
TWA MAP (mmHg)	0.35 (0.82)	0.0	8.75

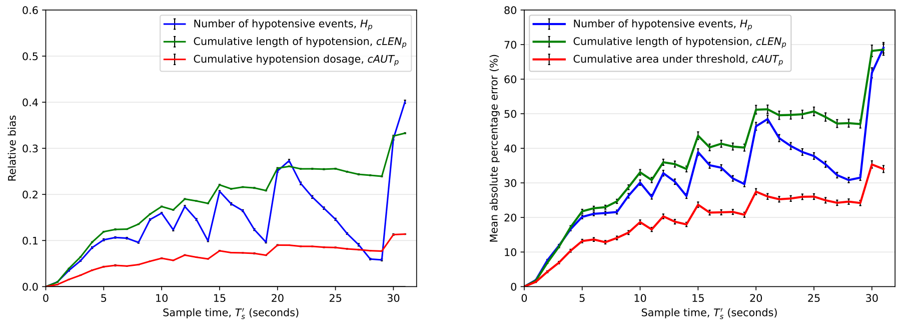

(a) (b)

Figure S10: VitalDB performance for different amount of downsampling on number of hypotensive events, cumulative length of hypotensive events, and cumulative area under curve. (a) Relative bias (Eq. 8) (b) Mean Absolute Percentage Error (MAPE)

set. It was done in the same way as explained in Section S1 and the results are shown in Table S5. The probability plots for the included VitalDB data set are shown in Fig. S11.

Table S5: Mean and median of the simulation and patient data for VitalDB

	$E[\tau_h]$	median(τ_h)	$E[\tau_n]$	median(τ_n)
Patient Data	13.3	2.26	95.9	2.59
Simulation	13.3	1.39	95.9	8.04

All values in seconds.

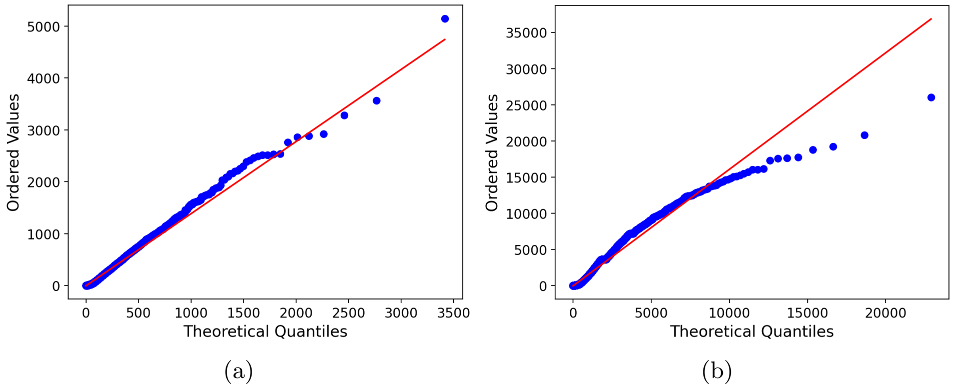

Figure S11: Probability plots of VitalDB data set for the chosen Weibull distributions. (a) Hypotension period length (τ_h), (b) In-between times (τ_n)

The parameters τ_h and τ_n are different in the VitalDB data set compared to the Karolinska data set, which is to be expected given the different patient cohort. However, not only the in-between times (τ_n) are longer, but also the hypotension period length τ_n are longer. This suggests that the basic modeling used where we only changed the in-between times (τ_n) to simulate different cohorts is somewhat too simplistic. Furthermore, from the probability plots in Figure S11, we can see that the match is still reasonable, but not as good as with the Karolinska data set in Figure S2. Nevertheless, all simulations are approximations of the reality and we feel that our simulations results in the article are valid.

Finally, we also tried the bias compensation approach on the VitalDB data set. It was done in the same fashion as with the Karolinska data set, and the VitalDB results are shown in Table S6 (Variant 1 only). It is again clear that different bias compensation (γ) is needed for the different metrics as before. We can note that the best γ values for VitalDB are the same as

Table S6: The effect of bias compensation, VitalDB, variant 1

H_p	$\gamma = 1$		$\gamma = 1/1.4$	
$\text{Bias}[\gamma\tilde{H}/H]$	0.687	(2.03)	-0.454	(1.904)
$\text{Bias}_p[\gamma\tilde{H}_p/H_p]$	0.346	(0.764)	-0.037	(0.536)
MAE	0.886	(1.95)	0.870	(1.753)
$cLEN_p$	$\gamma = 1$		$\gamma = 1/1.4$	
$\text{Bias}[\gamma c\tilde{LEN}/cLEN]$	2.99	(6.47)	-1.73	(6.92)
$\text{Bias}_p[\gamma c\tilde{LEN}_p/cLEN_p]$	0.433	(0.844)	0.0161	(0.5971)
MAE	3.01	(6.46)	2.67	(6.62)
$cAUT_p$	$\gamma = 1$		$\gamma = 1/1.2$	
$\text{Bias}[\gamma c\tilde{AUT}/cAUT]$	6.64	(21.2)	-8.79	(36.68)
$\text{Bias}_p[\gamma c\tilde{AUT}_p/cAUT_p]$	0.221	(0.598)	0.0143	(0.4940)
MAE	7.25	(21.0)	11.8	(35.8)

the ones for Karolinska. However, this does not imply that the optimal bias compensation parameter (γ) is independent of the patient cohort. Fig. 5 in the article suggests otherwise. However, it could be true that the best bias compensation (γ) depends much more on the L and T'_s combination as well as Variant 1 or 2 than on the patient cohort.

S8 Excluded Patients

We excluded patients from our data set with less than 90% available ABP data due to lack of good quality data. This corresponded to $n = 38$ or 8.3% of the patients in the Karolinska data set and $n = 155$ or 7.3% of the VitalDB patients. In Table S7, we list the patient characteristics for these excluded patients. Note that calculating the hypotension metrics for these patients is not possible due to lack of sufficient ABP data. The mean MAP is calculated over the available MAP data only.

Table S7: Excluded Patient Demographics. SD is standard deviation.

	Mean (SD) or N (%)	Min	Max
Karolinska data set ($N = 38$)			
Age (years)	67.42 (12.63)	23	91
sex, Male	16 (42.1%)		
Surgery length (hours)	4.85 (2.08)	2.0	10.07
Available ABP data (hours)	3.09 (2.04)	0.31	7.68
Mean MAP (mmHg)	74.54 (5.72)	62.38	86.6
VitalDB data set ($N = 155$)			
Age (years)	59.32 (15.39)	18.0	89.0
Sex, Male	88 (56.8%)		
Surgery length (hours)	4.66 (2.19)	2.0	15.92
Available ABP data (hours)	3.44 (1.82)	0.07	12.98
Mean MAP (mmHg)	79.12 (11.21)	53.52	106.99

References

[1] V. Salmasi, K. Maheshwari, D. Yang, E. J. Mascha, A. Singh, D. I. Sessler, and A. Kurz, "Relationship between Intraoperative Hypotension, Defined by Either Reduction from Baseline or Absolute Thresholds, and Acute Kidney and Myocardial Injury after Noncardiac Surgery: A Retrospective Cohort Analysis," *Anesthesiology*, vol. 126, pp. 47–65, Jan. 2017.

[2] L. Y. Sun, D. N. Wijeysundera, G. A. Tait, and W. S. Beattie, "Association of Intraoperative Hypotension with Acute Kidney Injury after Elective Noncardiac Surgery," *Anesthesiology*, vol. 123, pp. 515–523, Sept. 2015.

[3] J. A. R. van Waes, W. A. van Klei, D. N. Wijeysundera, L. van Wolfswinkel, T. F. Lindsay, and W. S. Beattie, "Association between Intraoperative Hypotension and Myocardial Injury after Vascular Surgery," *Anesthesiology*, vol. 124, pp. 35–44, Jan. 2016.

[4] H.-C. Lee, Y. Park, S. B. Yoon, S. M. Yang, D. Park, and C.-W. Jung, "VitalDB, a high-fidelity multi-parameter vital signs database in surgical patients," *Scientific Data*, vol. 9, p. 279, June 2022.
